# *AAC* as a Potential Target Gene to Control *Verticillium dahliae*

**DOI:** 10.3390/genes8010025

**Published:** 2017-01-10

**Authors:** Xiaofeng Su, Latifur Rehman, Huiming Guo, Xiaokang Li, Rui Zhang, Hongmei Cheng

**Affiliations:** Biotechnology Research Institute, Chinese Academy of Agricultural Sciences, Beijing 100081, China; suxiaofeng@caas.cn (X.S.); latif_ibge@yahoo.com (L.R.); guohuiming@caas.cn (H.G.); lixiaokang2016@163.com (X.L.)

**Keywords:** *Verticillium dahliae*, *VdAAC*, RNAi, growth, virulence

## Abstract

*Verticillium dahliae* invades the roots of host plants and causes vascular wilt, which seriously diminishes the yield of cotton and other important crops. The protein AAC (ADP, ATP carrier) is responsible for transferring ATP from the mitochondria into the cytoplasm. When *V. dahliae* protoplasts were transformed with short interfering RNAs (siRNAs) targeting the *VdAAC* gene, fungal growth and sporulation were significantly inhibited. To further confirm a role for *VdAAC* in fungal development, we generated knockout mutants (Δ*VdACC*). Compared with wild-type *V. dahliae* (Vd wt), Δ*VdAAC* was impaired in germination and virulence; these impairments were rescued in the complementary strains (Δ*VdAAC-C*). Moreover, when an RNAi construct of *VdAAC* under the control of the 35S promoter was used to transform *Nicotiana benthamiana*, the expression of *VdAAC* was downregulated in the transgenic seedlings, and they had elevated resistance against *V. dahliae*. The results of this study suggest that *VdAAC* contributes to fungal development, virulence and is a promising candidate gene to control *V. dahliae*. In addition, RNAi is a highly efficient way to silence fungal genes and provides a novel strategy to improve disease resistance in plants.

## 1. Introduction

*Verticillium dahliae* is one of the most destructive soil-borne fungi, infecting many important economic crops, fruit trees and ornamental flowers [1,2]. This fungus can cause typical disease symptoms, including stunted growth, necrosis, wilt and defoliation, which severely decrease the yield and quality of crops [3]. Each year, *Verticillium* wilt is reported to cause extensive economic losses to the crop industry [4]. The fungus can survive in soil for many years and infect the roots of its hosts. Its mycelium then abundantly colonizes the vascular bundle to block the transportation of nutrients [5]. Once the fungus is established in the host, *Verticillium* wilt is an intractable disease because of the intricate pathogenic mechanism of *V. dahliae* [6]. Currently, no fungicide is available to cure the disease caused by this fungus [7]. Previous studies on *V. dahliae* have thus focused on identifying genes that are crucial for fungal development and virulence [8,9,10], inestimable knowledge for crop breeding programs.

RNA interference (RNAi) is an effective tool to investigate gene function and elevate plant resistance against a fungus [11,12,13]. In *V. longisporum*, the expression of *Vlaro2* was reduced via RNAi, resulting in a bradytrophic mutant [14]. In vitro cultures of *Fusarium graminearum*, the introduction of double-stranded (ds)RNA that targeted cytochrome P450 lanosterol C14α-demethylase-encoding genes inhibited fungal growth. Similarly, expressing the same region of dsRNA into susceptible *Arabidopsis thaliana* and *Hordeum vulgare* conferred high resistance to fungal infection [15]. Transgenic banana plants with siRNAs targeted against velvet and *Fusarium* transcription factor 1 were protected against *Fusarium oxysporum* f. sp. *cubense* (Foc) [16], as were transgenic cotton plants against *V. dahliae* when the fungal *VdH1* gene was silenced [17].

Genes for essential cellular components may prove to be likely effective targets. For example, mitochondrial carriers are a series of proteins that transport nucleotides, amino acids, fatty acids, and so on across the inner mitochondrial membrane of eukaryotes [18]. Of these carriers, the highly conserved AAC is the most abundant protein [19,20]. AAC is essential for maintaining fluxes in energy and mediating the exchange of ADP and ATP between the mitochondria and cytoplasm [21]. AAC consists of six transmembrane helices embedded in the inner mitochondrial membrane with its N- and C-terminals exposed to the cytosolic side [22]. The C-terminal structure of yeast AAC is predicted to be involved in regulating the accessibility of the transmembrane core to water [23]. Silencing of the *AAC* gene of *Blumeria graminis* by biolistically bombarding barley cells with RNAi constructs led to the formation of fewer haustoria in barley cells [24]. In *Saccharomyces cerevisiae*, AAC might transmit a signal and facilitate permeabilization of the outer mitochondrial membrane to accelerate mitochondrial degradation followed by cytochrome C release during acetic acid-induced apoptosis [25]. Thylakoid ADP/ATP carrier (TAAC), apart from regulating ADP and ATP balance, has an additional role in transporting 3′-Phosphoadenosine 5′-phosphosulfate as the high-energy sulfate donor through the plastid envelope in Arabidopsis [26]. AAC also increases mitochondrial proton conductance for adapting to cold water stress in king penguins [27]. A decrease in the expression of *Trypanosoma brucei* AAC resulted in a reduced level of cytosolic ATP and mitochondrial oxygen consumption, severe growth defects and elevation in the amount of reactive oxygen species [28].

Although the functions of the *AAC* gene in development, resistance and signal transduction pathways have been explored in other species, its role in the development and virulence of *V. dahliae* has not yet been reported. In the present study, we used siRNA-induced silencing of the *VdAAC* gene in *V. dahliae* to establish the relationship between *VdAAC* and fungal development. Deletion of *VdAAC* resulted in reduced colony growth and sporulation. Virulence was significantly decreased in the Δ*VdAAC* mutants compared with the wild type (Vd wt) and complemented strains (Δ*VdAAC-C*). Confocal microscopic observations revealed that conidial germination of Δ*VdAAC* was significantly impaired. Moreover, transgenic *N. benthamiana* expressing dsRNA against *VdAAC* showed strong resistance against *V. dahliae*. Our results indicate that *VdAAC* contributes to fungal germination, development and sporulation, which are requisite for the fungus to invade the plants and induce full virulence in the host. For potential exploitation of this gene to protect crops against *V. dahliae*, its biological function needs to be further elaborated.

## 2. Materials and Methods

### 2.1. Fungal Strains, Plants and Inoculation with V. dahliae

Strain V991, a highly toxic and defoliating wild-type pathogenic strain of *V. dahliae*, was kindly gifted by Prof. Guiliang Jian of the Institute of Plant Protection, Chinese Academy of Agricultural Sciences (CAAS). *V. dahliae* strain Vd-GFP that expresses *GFP*, is from our laboratory culture collection [29]. Single-conidium cultures of all *V. dahliae* strains were grown in complete medium broth (CM) at 25 °C. After 1 week, the conidia were harvested for inoculation.

Two-week-old seedlings of *N. benthamiana* were transplanted from Murashige-Skoog (MS) agar into disinfested soil and incubated in the greenhouse at 23 ± 2 °C, 75% ± 5% relative humidity, and a photoperiod of 16 h day/8 h night. After 1 month, seedlings with 6–8 leaves were inoculated by dipping the roots in a suspension of 10^6^ conidia·mL^−1^ for 2 min.

### 2.2. Disease Index

Disease severity was evaluated using a five-grade scale based on a previous study with modifications [30]: grade 0, no wilt; grade 1, less than two leaves wilting; grade 2, three to five leaves wilting; grade 3, more than five leaves wilting or chlorotic; and grade 4, plant death or near death. Each respective experiment comprised 5 seedlings and was independently repeated three times for each assessment. Symptoms were recorded and the disease index (DI) calculated at 10 days post inoculation (dpi), 11 dpi and 12 dpi using the formula: DI = [Ʃ (number × grade)/(5 × 4)] × 100 [30,31].

### 2.3. Bioinformatics Analysis

The whole sequence of *VdAAC* was obtained from the *Verticillium* genomic database (www.broadinstitute.org). Amino acid sequences homologous to AAC in other species, identified with a Blastp search of the Protein Data Base (PDB), were used to construct a phylogenetic tree in MEGA software (version 6.06) (http://www.megasoftware.net/).

### 2.4. siRNA Design and Transformation of V. dahliae Protoplasts

The siRNAs targeting different regions of the *VdAAC* gene (siRNA-1, siRNA-2, siRNA-3 and siRNA-4) were designed using BLOCK-iT™ (Invitrogen, Carlsbad, CA, USA) RNAi Designer and synthesized by Oligobio, Beijing, China. The siRNAs sequences are given in Table 1, and the locations of these siRNAs in *VdAAC* are displayed in Figure S1A.

*V. dahliae* protoplasts isolated from fresh mycelia were transformed with the siRNAs as described in our previous study [29]. After 72 h in TB3 broth, the mycelia were collected to extract RNA with an RNA Extraction Kit (YPHBio, Tianjin, China). First strand cDNA was synthesized using a Reverse Transcription Kit (TransGen, Beijing, China) based on the manufacturer’s instructions. qRT-PCR was carried out with a 7500 Real Time PCR System (ABI, Foster City, CA, USA) [29]. *Vdactin* was used as a housekeeping gene [32]. The relative expression level of *VdAAC* was analyzed using the 2^−ΔΔCt^ method. The standard curve met the experimental requirements (R^2^ > 0.99, E > 95%) [33]. Transformed protoplasts were also cultured for 2 weeks on the center of PDA (Potato Dextrosa Agar: potato infusion 200 g, dextrose 20 g and agar 20 g in 1 L H_2_O) plates to measure colony diameter and count the conidia produced to assess the effect of silencing on fungal growth and sporulation.

### 2.5. Plasmid Construction and Fungal Transformation

For creating a knockout-infused gene fragment, flanking regions (1 kbp upstream and downstream) of the *VdAAC* gene and a hygromycin resistance (HPT) expression cassette were amplified and fused via the overlapping sequences.

For *GFP* (Green Fluorescence Protein) disruption mutants (Δ*VdAAC-GFP*), the neomycin resistance (Neo^R^) cassette containing XbaI and BstEII restriction sites was cloned into the pCAMBIA1302 vector. After that, the *GFP* expression cassette was introduced into the plasmid via XbaI and KpnI restriction sites to generate pCAMBIA1302::Neo::GFP. Meanwhile, the *VdAAC* ORF was substituted for the *GFP* open reading frame (ORF) of the recombinant plasmid as pCAMBIA1302::Neo::VdAAC for complementary strains.

The respective constructs (knockout-infused fragment, pCAMBIA1302::Neo::GFP, pCAMBIA1302::Neo::VdAAC) were used to transform *V. dahliae* protoplasts [29]. Transformants were selected based on antibiotic resistance and confirmed by RT-PCR. The primers are listed in Table 2.

### 2.6. Stress Treatments of V. dahliae Strains

For characterizing the development and morphology of the wild type and mutant strains of *V. dahliae*, 10 µL samples of 1 × 10^6^ conidia·mL^−1^ of the respective strains were cultured on Czapek-Dox (3 g NaNO_3_, 1 g K_2_HPO_4_, 0.5 g MgSO_4_.7H_2_O, 0.5 g KCl, 0.01g FeSO_4_ and 30 g Sucrose in 1 L H_2_O) agar with either 0.5 M NaCl or sorbitol. Similarly, plates containing conidia of the respective strains were exposed to UV light for 10 s in a Gel doc system (Syngene, Cambridge, UK) to assess the impact of UV-stress on the survival of these conidia [34]. The plates were then incubated at 25 °C and the colony diameter on each plate was measured after 2 weeks. For estimating conidia production, 3 mL of sterilized water was added to each plate, which was then gently shaken to release the conidia [35]. The conidia were then counted using a light microscope (BX52, OLYMPUS, Tokyo, Japan).

### 2.7. Confocal Microscopy

Confocal microscopy was used to facilitate the observation of the infection process of both wild type and mutant strains. *N. benthamiana* seedlings were inoculated with Vd-GFP and Δ*VdAAC-GFP* strains respectively. At 7 dpi, roots of the infected plants were collected, washed with water for 3 times and then observed under confocal microscope (LSM 700, Carl Zeiss, Jena, Germany) [36].

### 2.8. Plasmid Construction and Plant Transformation

A pair of primers was designed based on the *VdAAC* ORF [37] (Table 2). The targeted fragment (648 bp) in *VdAAC*, shown in Figure S2A, was amplified with partial BP adaptors. The whole sequence was cloned using BP site primers and inserted into pDONR207 by a BP recombinant reaction (Invitrogen, Carlsbad, CA, USA). Then, the targeted fragment was cloned into the pK7GWIWG2(I) vector using an LR recombinant reaction (Invitrogen, Carlsbad, CA, USA). The recombinant plasmid was named pK7GWIWG2(I)-VdAAC (Figure S2B), confirmed by sequencing, and then used to transform *Agrobacterium tumefaciens* strain LBA4404 using electroporation [38].

Sterile leaves of *N. benthamiana* were immersed in *A. tumefaciens* strain LBA4404 containing the recombinant plasmid and transferred to MS agar. After 3 days, the leaves were cultured on MS agar containing 100 mg·L^−1^ kanamycin [39]. Seedlings were confirmed by PCR to be transgenic (Figure S2C). Two transgenic lines (Trans-1 and Trans-2), expressing dsRNA against *VdAAC* were selected for further analysis. The primer sequences for detection are listed in Table 2.

### 2.9. Analysis of Fungal Biomass

Colonization of *V. dahliae* in seedlings was quantified at 12 dpi by isolating DNA from the roots, stems (0–3 cm above the soil line) and leaves, respectively, using the Plant Genomic DNA Kit (TIANGEN, Beijing, China). Fungal biomass was quantified via qRT-PCR by amplifying ITS1 and ITS2 of rDNA (Z29511) of *V. dahliae* [35]. The *N. benthamiana* housekeeping gene (*Nbactin*) was used as an internal control [40]. The primers are listed in Table 2.

### 2.10. qRT-PCR Analysis of the Expression Level of Targeted Genes

The silencing effect of *VdAAC* in the infected seedlings was assessed by extracting RNA from roots at 12 dpi for *qRT-PCR* as described in Section 2.4. The housekeeping gene *Vdactin* was used as a control [32]. The relative expression of targeted gene was analyzed using the 2^−∆∆Ct^ method. The standard curve met experimental requirements (R^2^ > 0.99, E > 95%) [33]. The primers are listed in Table 2.

### 2.11. Statistical Analysis

All experiments were independently repeated thrice, and data was analyzed for significant differences among the groups using Duncan’s multiple range test (*p* < 0.05) and SPSS Statistics 17.0 software (SPSS, Chicago, IL, USA).

## 3. Results

### 3.1. Bioinformatics Analysis of VdAAC

**Figure 1:**
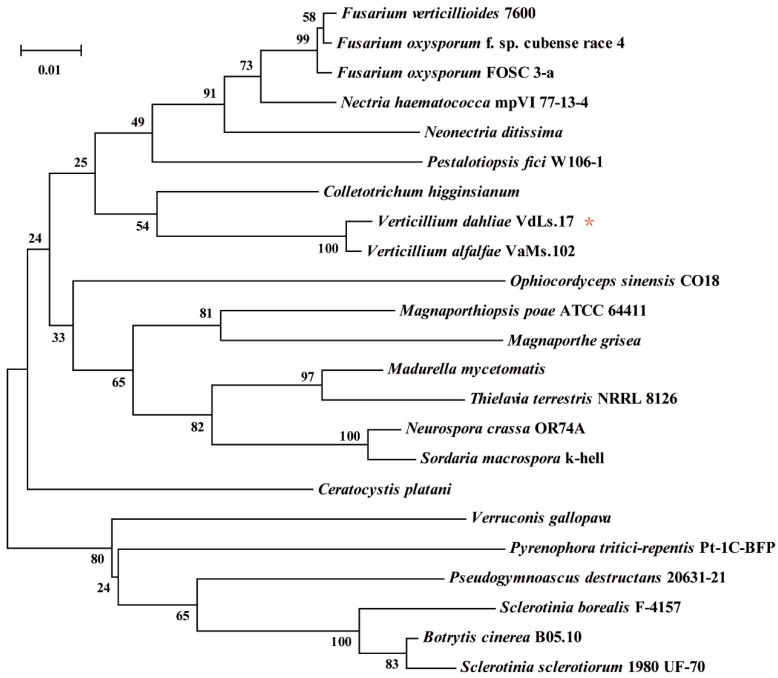
Phylogenetic analysis of AAC amino acid sequences from different fungal species. The phylogenetic tree was constructed by MEGA software (version 6.06; bootstraps: 1000). Fungal species and protein accession numbers: *Verticillium dahliae* VdLs.17 (XP_009654735.1); *Nectria haematococca* mpVI 77-13-4 (XP_003051617.1); *Fusarium oxysporum* FOSC 3-a (EWZ02370.1); *Verticillium alfalfae* VaMs.102 (XP_003004480.1); *Fusarium verticillioides* 7600 (EWG42987.1); *Pestalotiopsis fici* W106-1 (XP_007835574.1); *Fusarium oxysporum* f. sp. *cubense* race 4 (EMT68221.1); *Magnaporthiopsis poae* ATCC 64411 (KLU91586.1); *Neonectria ditissima* (KPM44251.1); *Madurella mycetomatis* (KOP45712.1); *Colletotrichum higginsianum* (CCF32866.1); *Ceratocystis platani* (KKF94862.1); *Pseudogymnoascus destructans* 20631-21 (XP_012739498.1); *Botrytis cinerea* B05.10 (XP_001559435.1); *Sclerotinia sclerotiorum* 1980 UF-70 (XP_001598713.1); *Verruconis gallopava* (KIW06207.1); *Sclerotinia borealis* F-4157 (ESZ96107.1); *Neurospora crassa* OR74A (XP_011393638.1); *Sordaria macrospora* k-hell (XP_003351160.1); *Pyrenophora tritici*-*repentis* Pt-1C-BFP (XP_001934086.1); *Ophiocordyceps sinensis* CO18 (EQK99145.1); *Magnaporthe grisea* (AAX07662.1); *Thielavia terrestris* NRRL 8126 (XP_003658188.1). * represents the query sequence.

The ORF of *VdAAC* (VDAG_07535.1) contains 930 bp, which encodes a protein with 310 amino acids (GenBank NO.: XP_009654735.1). The neighbor-joining phylogenetic tree for the *VdAAC* sequences from *V. dahliae* and other fungi constructed using MEGA (bootstraps: 1000) demonstrated that the AAC sequences are relatively conserved among these fungal species (Figure 1).

### 3.2. Silencing of VdAAC Effectively Inhibited Fungal Growth and Sporulation

**Figure 2:**
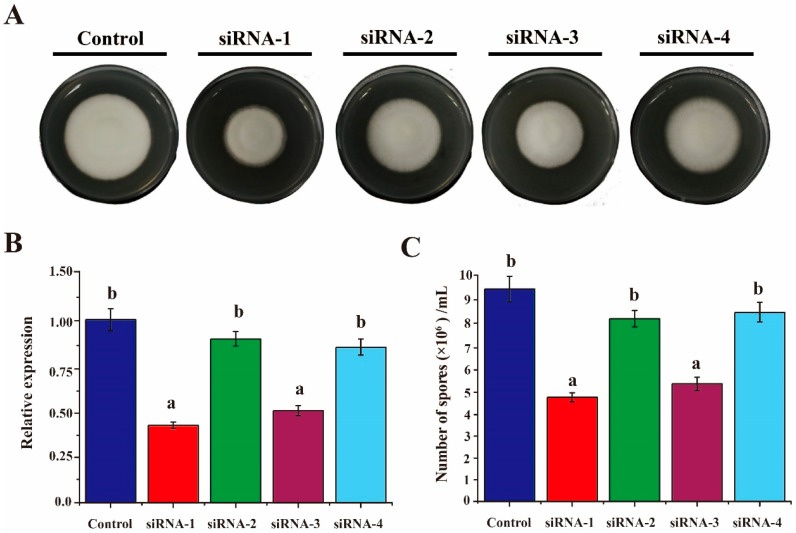
Assay for siRNA inhibition of the *VdAAC* gene. *V. dahliae* protoplasts were transformed with 10 μM siRNA-1, siRNA-2, siRNA-3, siRNA-4 or control, respectively. After regenerating for 18 h in TB3 broth, the protoplasts were cultured in the center of a PDA plate. (**A**) Colony morphology on PDA after 2 weeks; (**B**) Relative expression levels of *VdAAC* in different RNAi-treated groups. RNA was extracted from mycelia 72 h after transformation. First strand cDNA was synthesized, and qRT-PCR was carried out; (**C**) Number of conidia produced by the control and siRNA groups after 2 weeks. Error bars represent standard deviation (SD) calculated from means for three independent replicates. Significant differences (*p* < 0.05) among means for the different incubation times in Duncan’s multiple range test are indicated with different letters.

In our previous study [29], we showed that the siRNAs can enter *V. dahliae* protoplasts to silence the targeted genes. Thus, siRNAs designed against *VdAAC* were used to transform the protoplasts. After 2 weeks, the mean colony diameter of the siRNA-1 group (14.2 mm) and siRNA-3 (16 mm) were distinctly smaller than that of the siRNA-control group (24.5 mm) (Figure 2A and Figure S1B). To further confirm whether the silencing of *VdAAC* gene led to the reduced colony growth, qRT-PCR was carried out to determine the relative expression level of *VdAAC* in all the groups. The data was consistent with the colony diameter assessment (Figure 2B). Similarly, the siRNA-1 and siRNA-3 groups produced fewer conidia than the other groups did (Figure 2C). Taken together, these results demonstrate that inhibition of *VdAAC* expression impairs the fungal growth and sporulation.

### 3.3. Generation of the VdAAC Mutant

**Figure 3:**
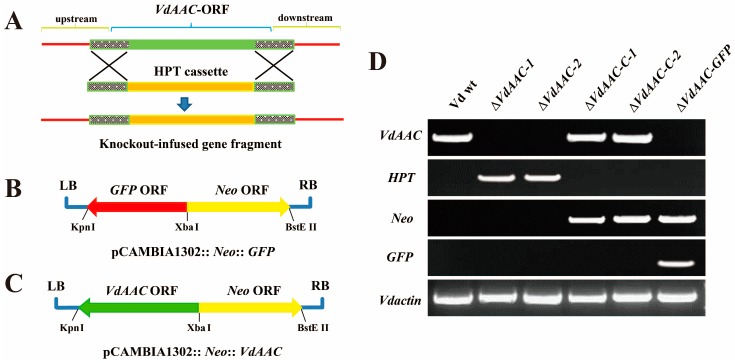
Disruption of the *VdAAC* gene and confirmation of *V. dahliae* mutants. (**A**) Construction of the knockout-infused fragment for gene disruption. The fragment was obtained by fusing about 1 kb upstream and downstream of the *VdAAC* gene and hygromycin resistance (HPT) cassette; (**B**) GFP expression cassette (GFP) and neomycin resistance (Neo) cassette were introduced into pCAMBIA1302 to generate pCAMBIA1302::Neo::GFP; (**C**) GFP expression cassette (GFP) was repalced by VdAAC expression cassette (VdAAC) to produce pCAMBIA1302::Neo::VdAAC; (**D**) Confirmation of transformants. RNA was isolated from mycelia of mutants cultured in CM broth. The first strand cDNA was synthesized, and RT-PCR was carried out to confirm the transformants. *Vdactin* gene was used as a housekeeping gene.

To further explore the function of *VdAAC*, we used the knockout-infused fragment to transform Vd wt protoplasts to generate the *VdAAC* deletion mutants (Figure 3A). pCAMBIA1302::Neo::GFP was used to transform the gene deletion strains to facilitate confocal microscopy while pCAMPIA1302::Neo::VdAAC for complementation assays (Figure 3B,C).

Subsequently, transformants of Δ*VdAAC* (*VdAAC* deletion mutant), Δ*VdAAC-C* (*VdAAC* complementation mutant, obtained from transforming pCAMPIA1302::Neo::VdAAC into Δ*VdAAC*) and Δ*VdAAC-GFP* (VdAAC mutant transformed with GFP plasmid) strains were selected randomly and anlayzed by PCR (Figure 3D). Two gene deletion strains Δ*VdAAC-1* and Δ*VdAAC-2* and two complementary strains Δ*VdAAC-C-1* (derived from the transformation of Δ*VdAAC-1*) and Δ*VdAAC-C-2* (derived from the transformation of Δ*VdAAC-2*) were selected for further work. As expected, *VdAAC* expression was only detected in Vd wt, Δ*VdAAC-C-1* and Δ*VdAAC-C-2*, and not in Δ*VdAAC-1* and Δ*VdAAC-2*. Moreover, *GFP* expression was detected in Δ*VdAAC-GFP*. The transformants were then further analyzed for the role of *VdAAC* in the development and virulence of *V. dahliae*.

### 3.4. Stress Response Assay

**Figure 4:**
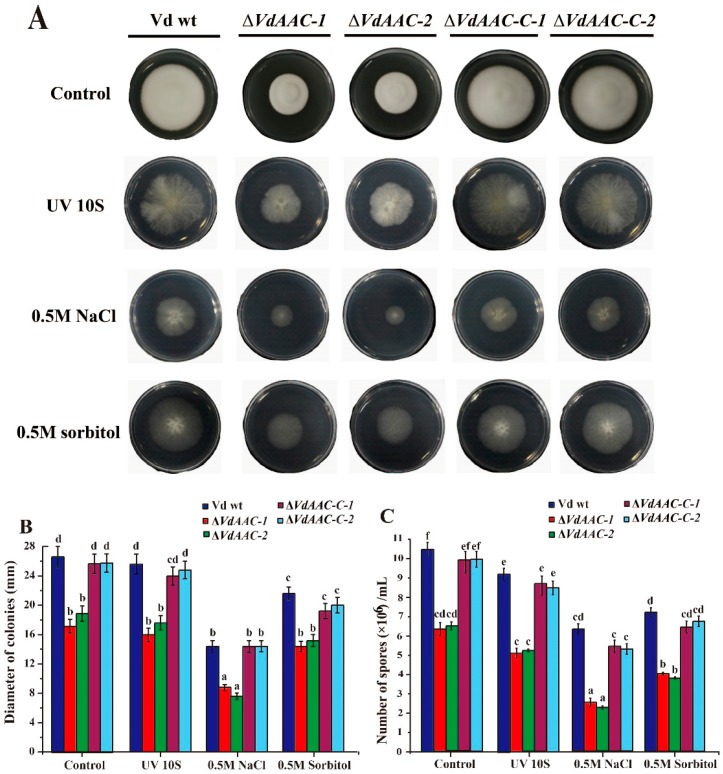
Colony morphology, diameter and conidia number of Δ*VdAAC*, Δ*VdAAC-C* and wild-type *V. dahliae* (Vd wt) strains exposed to stresses and without stress. Conidia from the respective strains were exposed to UV light and cultured in the center of Czapek-Dox agar plates. Conidia without UV light exposure were cultured on media supplemented with either NaCl or sorbitol. After 2 weeks, fungal traits were assessed: (**A**) colony morphology; (**B**) colony diameter; and (**C**) conidia number of mutants and Vd wt strain. Different letters (a–f) above the bars represent significant differences among the treatment groups (*p* < 0.05) as determined by the Duncan’s multiple range test.

The function of *VdAAC* in stress responses was analyzed by exposing conidia of Vd wt, Δ*VdAAC*, and Δ*VdAAC-C* strains to UV light, high NaCl or sorbitol. The phenotype of all the strains in the absence of stress was much better (Figure 4A). Exposure to each stress resulted in no significant reduction in the colony diameters and conidial number of Δ*VdAAC* when compared with the effect of the stress on Vd wt and Δ*VdAAC-C* (Figure 4A,B). Although the effect of NaCl and sorbitol stresses was significant on the growth of all the strains, however the ration of growth reduction for gene deletion mutants with wild type and complementary strains was similar to no stress conditions (Figure 4C). In brief, *VdAAC* might not have a significant contribution in stress tolerance.

### 3.5. Expression of Genes Involved in Energy Metabolism in ΔVdAAC

**Figure 5:**
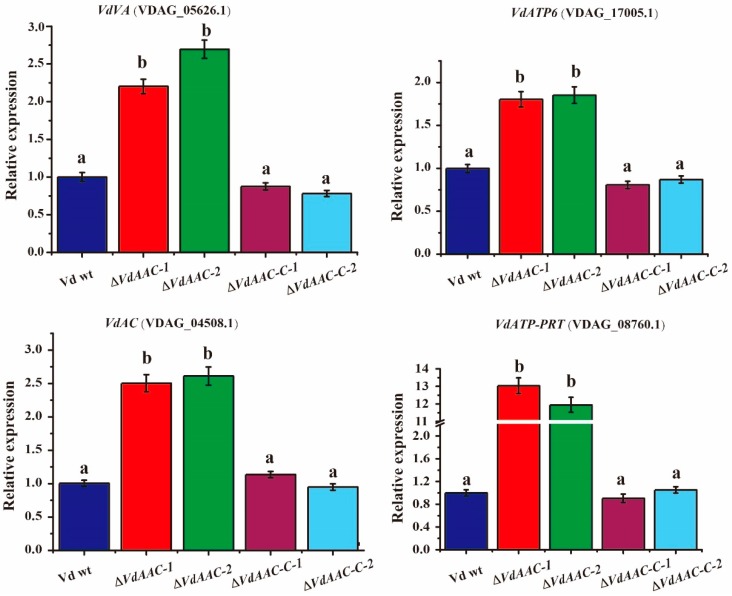
Relative expression of related genes involved in energy metabolism. Conidia from the respective strains were cultured in CM broth. After 4 days, the mycelia were collected for RNA extraction and the cDNA was synthesized. Expression patterns of four genes, vacuolar ATPase (VDAG_05626.1, *VdVA*), ATP synthase F0 subunit 6 (VDAG_17005.1, *VdATP6*), adenylate cyclase (VDAG_04508.1, *VdAC*), ATP phosphoribosyltransferase (VDAG_08760.1, *VdATP-PRT*), were determined by qRT-PCR. *Vdactin* gene was used as the reference gene for the expression analysis. Significant differences among the treatment groups (*p* < 0.05) are indicated by different letters (a, b) above the bars determined by the Duncan’s multiple range test.

To understand the regulation of target genes relevant to energy metabolism, we analyzed the expression of vacuolar ATPase (VDAG_05626.1, *VdVA*), a gene that is involved in generating electrochemical potential across the vacuolar membrane [41,42,43], ATP synthase F0 subunit 6 (VDAG_17005.1, *VdATP6*), responsible for phosphorylating ADP [44,45], adenylate cyclase (VDAG_04508.1, *VdAC*), required for the conversion of ATP to cAMP [46,47] and ATP phosphoribosyltransferase (VDAG_08760.1, *VdATP*-*PRT*), necessary for the formation of phosphoribosyl-ATP and inorganic pyrophosphate [48,49,50,51] in Vd wt, Δ*VdAAC* and Δ*VdAAC-C*. The expression of these genes in Δ*VdAAC* strains increased >2-fold as compared with Vd wt and Δ*VdAAC-C* (Figure 5). Collectively, these results suggest that the upregulation of these genes might be due to the disturbance in ADP/ATP levels.

### 3.6. VdAAC Is Involved in Fungal Virulence

**Figure 6:**
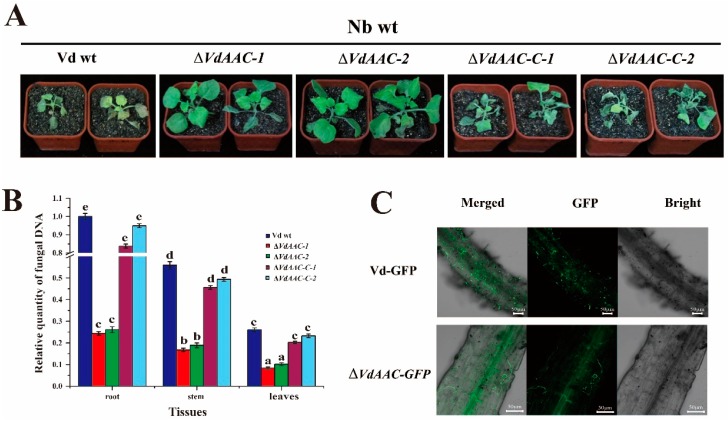
Virulence analysis of *ΔVdAAC*, *ΔVdAAC*-*C* and wild-type *V. dahliae* (Vd wt) strains on the wild-type *Nicotiana benthamiana* (Nb wt). The Nb wt seedlings were inoculated with 10^6^ conidia·mL^−1^. (**A**) Virulence phenotypes of Nb wt seedlings and (**B**) fungal biomass of different tissues of plants at 12 days after inoculation with the different strains. For the relative quantitative analysis, ITS1 and ITS2 of rDNA (Z29511) of *V. dahliae* were quantified relative to *N. benthamiana* housekeeping gene (*Nbactin*) for equilibration. (**C**) GFP fluorescence detection in roots of plants 7 days after inoculation with *Vd-GFP* or Δ*VdAAC-GFP*. Duncan’s multiple range test was applied to determine significant differences among the treatment groups (*p* < 0.05) indicated by different letters (a–e).

For evaluating the consequences of *VdAAC* deletion on fungal virulence, wild-type *N. benthamiana* (Nb wt) seedlings were inoculated with Vd wt, Δ*VdAAC*, and Δ*VdAAC-C*. At 12 dpi, seedlings inoculated with the Vd wt exhibited typical wilting symptoms and seemed nearly dead. In contrast, the disease index of seedlings inoculated with Δ*VdAAC* remained at a low level and was 70%–80% lower than that of the Vd wt group. The lower leaves of plants displayed a mild necrosis. The symptoms and disease index of seedlings inoculated with Δ*VdAAC-C* were similar to that of the Vd wt group (Figure 6A and Figure S3A). Fungal biomass in the various tissues of the plants inoculated with the different strains was then analyzed using qRT-PCR (Figure 6B). Fungal biomass of Δ*VdAAC* was significantly lower than that of the Vd wt and Δ*VdAAC*. These results were consistent with the phenotype and disease index data.

To investigate the *VdAAC* effect on fungal germination, 10^3^ conidia of Vd-GFP and Δ*VdAAC-GFP* strains were added to PDA plates. After 48 h, the germination of Δ*VdAAC-GFP* conidia was nearly half that of Vd-GFP (Figure S3B). Furthermore, when the infection process of Vd-GFP and Δ*VdAAC-GFP* strains was examined microscopically (Figure 6C), at 7 dpi, many conidia of Vd-GFP had germinated, and hyphae were growing on the root surface. In contrast, fewer Δ*VdAAC-GFP* conidia had germinated compared with Vd-GFP. Meanwhile, the fungal biomass was lower on the root surface. All these results demonstrate that *VdAAC* contributes to fungal germination and growth, requisite for invasion and full virulence.

### 3.7. DsRNA of VdAAC Confers Resistance against Vd in Transgenic Lines

**Figure 7:**
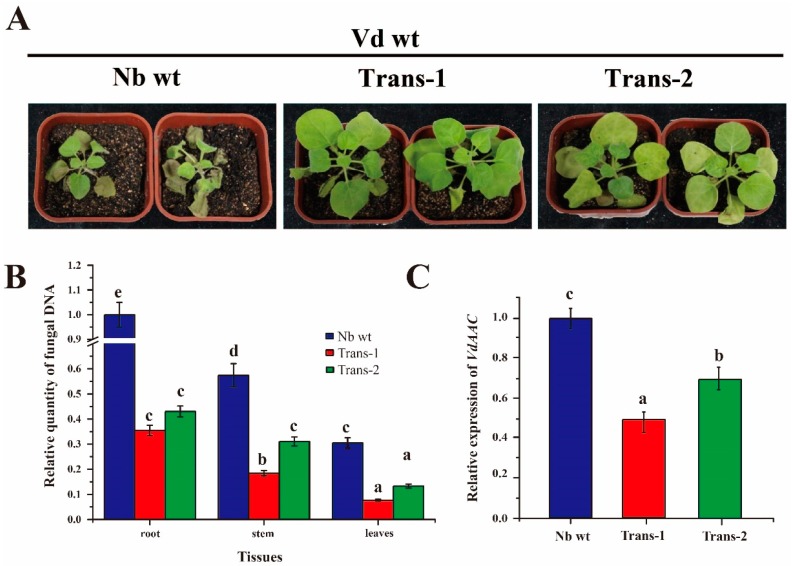
Assessment of the transgenic *Nicotiana benthamiana* for resistance against wild-type *V. dahliae*. One-month-old wild-type *Nicotiana benthamiana* (Nb wt) and transgenic lines (Trans-1 and -2) seedlings were inoculated with Vd wt and analyzed at 12 dpi. (**A**) Phenotypes of seedlings; (**B**) fungal biomass and (**C**) relative expression level of *VdAAC* determined by qRT-PCR. The bars with different letters (a–e) are significantly different (*p* < 0.05), based on the Duncan’s multiple range test.

Transgenic seedlings (Trans-1 and Trans-2) were inoculated with fungal Vd wt conidia to validate whether dsVdAAC can confer resistance against *V. dahliae* (Figure 7A and Figure S2D). From 10 dpi, the Nb wt group displayed typical wilt symptoms, and the disease index was more than 80. At 12 dpi, the seedlings of the Nb wt group were nearly dead, and the disease index was approximately 100. In contrast, at 10 dpi, seedlings of the transgenic groups had weak symptoms. At 12 dpi, the disease index was 70% lower in the Trans-1 group and 36% lower in the Trans-2 group than in the Nb wt group.

On the basis of the qRT-PCR to estimate fungal biomass in the root, stem and leaves of different groups at 12 dpi (Figure 7B), fungal biomass was significantly lower in transgenic seedlings than in Nb wt. To further examine whether the decreasing disease index in transgenic seedlings resulted from the silencing of *VdAAC*, we used qRT-PCR to analyze the relative expression level of *VdAAC* in transgenic and wild-type seedlings (Figure 7C). With the expression level of *VdAAC* in the Nb wt group estimated as 1, the Trans-1 group had better silencing efficiency (up to 47%) compared with 29% in Trans-2 group. The relative quantitative results, including the level of *V. dahliae* biomass and *VdAAC* expression, were strongly in accordance with the phenotypes of different groups.

## 4. Discussion

Because the membrane protein AAC is needed to maintain a balance between ADP and ATP to generate energy for cells, we postulated that siRNAs designed against the *VdAAC* gene could be introduced into *V. dahliae* to decrease the expression of *VdAAC*; silencing of *VdAAC* could ultimately inhibit the mycelial growth and sporulation. By transforming fungal protoplasts in this way, we confirmed this hypothesis. Further support for this data comes from a previous study in which the silencing of a single functional *AAC* gene (*TbAAC*), in *Trypanosoma brucei*, by RNAi resulted in a severe growth defect, mainly due to reduced mitochondrial ATP synthesis [28]. Consistent with our RNAi results, the Δ*VdAAC* mutant had significant reduced colony diameter, conidia number and virulence as compared with Vd wt and Δ*VdAAC-C*.

Previous studies indicated that genes related to energy metabolism are upregulated in response to adverse environments [52,53,54]. In litchi fruit, when exposed to cold temperature, short-term anaerobic and pure oxygen conditions, genes related to energy metabolism including *LcAAC* were found to be upregulated [55]. Under reduced oxygen tension, the *AAC* gene deletion mutants of *Schizosaccharomyces pombe* exhibited impaired growth and were also unable to grow on a nonfermentable carbon source [56]. In our study, however, we found that *VdAAC* gene does not have similar role of adapting the fungus to adverse conditions. When exposed to UV light and high osmotic stress, the ratio of reduction in mycelial growth and sporulation for Δ*VdAAC* with Vd wt and Δ*VdAAC-C* strains were not significant from no stress conditions. Overall, the gene deletion mutants (Δ*VdAAC*) grows at roughly 60% of the wild type (Vd wt) and complementary strains (Δ*VdAAC-C*) rate under both stress and no stress conditions.

The *AAC* gene has a vital role in maintaining ADP/ATP balance in vivo, transporting ATP to the cytoplasm and ADP to mitochondria [57,58]. The movement of adenine-nucleotide (ADP/ATP) across the inner membrane of mitochondria is dependent on the concentration of internal ATP and on the energy state of mitochondria. The knockout of this gene can have a significant effect on genes that are putatively involved in energy metabolism e.g., vacuolar ATPase (VDAG_05626.1, *VdVA*) was upregulated in gene deletion mutants. The deletion of *AAC* gene also had a significant impact on the expression ATP synthase F0 subunit 6 (VDAG_17005.1, *VdATP6*). Similarly adenylate cyclase (VDAG_04508.1, *VdAC*) and ATP phosphoribosyltransferase (VDAG_08760.1, *VdATP-PRT*), were also upregulated in mutants as compared to the wild type and complementary strains.

Under favorable conditions, conidia germinate and produce a germ tube as the first step to invade a host and initiate wilt disease [59]. As we discussed earlier, sporulation is also a requisite factor for this fungus to infect the host [60]. The genes involved in germination and sporulation have become a target to control this fungus [61]. Sporulation is significantly impaired in *VdPR3* deletion mutants, which had decreased virulence on cotton [62]. The disruption of *VdRNS/ER* downregulated glycan synthesis, leading to the inhibition of conidia germination and infection by *V. dahliae* [63]. In this study, germination and sporulation were significantly reduced in Δ*VdAAC*, suggesting these reductions were the main reason for reduced fungal biomass and virulence in Nb wt.

Transgenic plants harboring dsRNA against appropriate target genes can have improved resistance [64,65]. Expression of 16D10 dsRNA in *Arabidopsis* improved resistance against the four major species of root-knot nematodes [66]. Transgenic tomato plants expressing a hairpin construct exhibited resistance against potato spindle tuber viroid infection [67]. Wheat plants transformed with RNAi constructs against three targeted fungal genes exhibited strong resistance against *Puccinia triticina* and thus a suppressed disease phenotype [36]. In our study, in the transgenic lines of *N. benthamiana* that expressed dsVdAAC, expression of the targeted gene and fungal biomass were reduced in the plant, which also had a lower disease index than Nb wt did.

## 5. Conclusions

In this study, the siRNAs transformed into *V. dahliae* protoplasts silenced *VdAAC*, and mycelial growth and sporulation were inhibited. Gene knockout mutants, as compared with wild-type and complementary strains, were impaired in mycelial growth, conidia production, stress tolerance and virulence. Moreover, the transgenic plants expressing ds*VdAAC* showed enhanced resistance against *V. dahliae*. Taken together, the present data demonstrates that *VdAAC* has potential as a target gene for an RNAi-based strategy to protect crops against *V. dahliae*.

## Figures and Tables

**Table 1:** siRNA sequences designed against VdAAC.

Name	Sense Sequence	Antisense Sequence
Control	UUCUCCGAACGUGUCACGUTT	ACGUGACACGUUCGGAGAATT
siRNA-1	UCAAGCUCCUCAUCCAGAATT	UUCUGGAUGAGGAGCUUGATT
siRNA-2	GCAACACUGCCAACGUCAUTT	AUGACGUUGGCAGUGUUGCTT
siRNA-3	GCUUUCCGUGACAAGUUCATT	UGAACUUGUCACGGAAAGCTT
siRNA-4	GCAUGUACGACUCCAUCAATT	UUGAUGGAGUCGUACAUGCTT

**Table 2:** Primers and their sequences used in this study.

Primers	Sequence
qRT-AAC	TTGCCGAGTGCTTCAAGCGTAC
	GGCGTAGTCGAGGGAGTAGACG
qRT-Vdactin	GGCTTCCTCAAGGTCGGCTATG
	GCTGCATGTCATCCCACTTCTTC
qRT-VdITS	CCGCCGGTCCATCAGTCTCTCTGTTTATAC
	CGCCTGCGGGACTCCGATGCGAGCTGTAAC
qRT-Nbactin	GGACCTTTATGGAAACATTGTGCTCAGT
	CCAAGATAGAACCTCCAATCCAGACAC
qRT-VA	GGGTATTCAGACCCTATTGGACG
	CGAACTTCTTGTACTCAGCCTCC
qRT-ATP6	CTAGACCAATTTGAAATAAGA
	AAAGATTCTTGGCTAATAGAT
qRT-VdAC	TCTCCATCGTCTTCACCGACATCA
	TCTGCACGGCGAAACACCACA
qRT-VdATP-PRT	CGACGCCAACGTGCGGTCCTACAA
	GCCCGAGAAGCTCGTGCCAAT
HPT expression cassette	TTGAAGGAGCATTTTTGGGC
	TTATCTTTGCGAACCCAGGG
ΔAAC	CTTGGTGAAGGAGAGCGTTGAAAGT
	GCCCAAAAATGCTCCTTCAATGACAAGTTCAAGGCCATGTTCGGC
	CCCTGGGTTCGCAAAGATAACTCCGTTGCTGGTATCGTTGTCTAC
	GGTTCCTCGTCGCTGTCAATGACC
Neo expression cassette	aat*TCTAGA*GTTTGCGGGCTGTCTTGACG
	ata*GGTCACC*TACCTGTGCATTCTGGGTAA
GFP expression cassette	ggc*TCTAGA*CTTTCGACACTGAAATACGTCG
	ata*GGTACC*GCATCAGAGCAGATTGTACTGAGAG
ΔAAC-C	aaa*AGTACT*ATGTCCGTCGAGAAGCAG
	aaa*CTGCAG*TTATTTGAAGGCCTTGCC
Trans-AAC	GGGGACAAGTTTGTACAAAAAAGCAGGCTGTGCTTCAAGCGTAC
	GGGGACCACTTTGTACAAGAAAGCTGGGTCCCTTGAAGAGAGAC
Det-trans	CGTCATCCGTTACTTCCCTACCCA
	AGACCGGCAATACCGTCAGAGGC
Det-GFP	CGACGTAAACGGCCACAAGTT
	TCTTTGCTCAGGGCGGACTGG
Det-AAC	GCGCCAGTTCAACGGTCTTGTCG
	TCACCAGAGGTCATCATCATGCGAC
Det-Neo	GTTGTCACTGAAGCGGGAAGGG
	GCGATACCGTAAAGCACGAGGAA
Det-HPT	TTCGACAGCGTCTCCGACCTGA
	AGATGTTGGCGACCTCGTATTGGG

Restriction enzyme sites are indicated using bold and italic fonts.

## Supplementary Materials

The following are available online at www.mdpi.com/2073-4425/8/1/25/s1, Figure S1: Position of siRNAs designed from different regions of the *VdAAC* gene of *V. dahliae* and colony diameter in different RNAi-treated groups. (A) Position of siRNAs along the *VdAAC* gene. siRNAs were designed and synthesized by Oligobio, Beijing, China; (B) Colony diameters of control and siRNA groups observed 2 weeks after transformation on PDA agar plates. Figure S2: Evaluation of resistance for wild-type (Nb wt) and transgenic *N. benthamiana* against *V. dahliae*. (A) Region (189–836 bp) of *VdAAC* gene was amplified and cloned into pK7GW1WG2(I) by LR recombination reaction. Numbers indicate nucleotide positions; (B) Schematic representation of the pK7GWIWG2(I)-VdAAC construction containing the sense and antisense partial ORF of VdAAC; (C) Confirmation of transgenic plants transformed with pK7GWIWG2(I)-VdAAC by PCR. The Nb wt seedling served as the negative control. (D) Disease index for seedlings from 10 to 12 days post inoculation with V. dahliae. Figure S3: Virulence and germination analysis of mutants and wild-type *V. dahliae* (Vd wt). (A) Disease index for *N. benthamiana* seedlings at 10 to 12 dpi with ΔVdAAC, ΔVdAAC-C and Vd wt; (B) Percentage germination of conidia produced by Vd-GFP or ΔVdAAC-GFP after 48 h on PDA.

## Abbreviations

The following abbreviations are used in this manuscript:

AAC	ADP, ATP carrier
siRNAs	short interfering RNAs
RNAi	RNA interference
CM	complete medium broth
MS	Murashige-Skoog
dpi	days post inoculation
DI	disease index
ORF	open reading frame

## References

[1] Klosterman S.J., Atallah Z.K., Vallad G.E., Subbarao K.V. (2009). Diversity, pathogenicity, and management of Verticillium species. Annu. Rev. Phytopathol..

[2] Pang J., Zhu Y., Li Q., Liu J., Tian Y., Liu Y., Wu J. (2013). Development of Agrobacterium-mediated virus-induced gene silencing and performance evaluation of four marker genes in *Gossypium barbadense*. PLoS ONE.

[3] Pegg G.F., Brady B.L. (2002). Verticillium Wilts.

[4] Wang Y., Liang C., Wu S., Zhang X., Tang J., Jian G., Jiao G., Li F., Chu C. (2016). Significant improvement of cotton Verticillium wilt resistance by manipulating the expression of gastrodia antifungal proteins. Mol. Plant.

[5] Tsror L., Levin A.G. (2003). Vegetative compatibility and pathogenicity of *Verticillium dahliae* Kleb. Isolates from Olive in Israel. J. Phytopathol..

[6] Duressa D., Anchieta A., Chen D., Klimes A., Garcia-Pedrajas M.D., Dobinson K.F., Klosterman S.J. (2013). RNA-seq analyses of gene expression in the microsclerotia of *Verticillium dahliae*. BMC Genom..

[7] Fradin E.F., Thomma B.P. (2006). Physiology and molecular aspects of *Verticillium* wilt diseases caused by *V. dahliae* and *V. albo-atrum*. Mol. Plant Pathol..

[8] Zhang D.D., Wang X.Y., Chen J.Y., Kong Z.Q., Gui Y.J., Li N.Y., Bao Y.M., Dai X.F. (2016). Identification and characterization of a pathogenicity-related gene *VdCYP1* from *Verticillium dahliae*. Sci. Rep..

[9] Xiong D., Wang Y., Tang C., Fang Y., Zou J., Tian C. (2015). VdCrz1 is involved in microsclerotia formation and required for full virulence in *Verticillium dahliae*. Fungal Genet. Biol..

[10] Tian L., Xu J., Zhou L., Guo W. (2014). VdMsb regulates virulence and microsclerotia production in the fungal plant pathogen *Verticillium dahliae*. Gene.

[11] Nakayashiki H., Hanada S., Nguyen B.Q., Kadotani N., Tosa Y., Mayama S. (2005). RNA silencing as a tool for exploring gene function in ascomycete fungi. Fungal Genet. Biol..

[12] Deshmukh R., Purohit H.J. (2014). siRNA mediated gene silencing in *Fusarium* sp. HKF15 for overproduction of bikaverin. Bioresour. Technol..

[13] Mumbanza F.M., Kiggundu A., Tusiime G., Tushemereirwe W.K., Niblett C., Bailey A. (2013). In vitro antifungal activity of synthetic dsRNA molecules against two pathogens of banana, *Fusarium oxysporum* f. sp. *cubense* and *Mycosphaerella fijiensis*. Pest Manag. Sci..

[14] Singh S., Braus-Stromeyer S.A., Timpner C., Tran V.T., Lohaus G., Reusche M., Knufer J., Teichmann T., von Tiedemann A., Braus G.H. (2010). Silencing of *Vlaro2* for chorismate synthase revealed that the phytopathogen *Verticillium longisporum* induces the cross-pathway control in the xylem. Appl. Microbiol. Biotechnol..

[15] Koch A., Kumar N., Weber L., Keller H., Imani J., Kogel K.H. (2013). Host-induced gene silencing of cytochrome P450 lanosterol C14alpha-demethylase-encoding genes confers strong resistance to *Fusarium* species. Proc. Natl. Acad. Sci. USA.

[16] Ghag S.B., Shekhawat U.K., Ganapathi T.R. (2014). Host-induced post-transcriptional hairpin RNA-mediated gene silencing of vital fungal genes confers efficient resistance against *Fusarium* wilt in banana. Plant Biotechnol. J..

[17] Zhang T., Jin Y., Zhao J.H., Gao F., Zhou B.J., Fang Y.Y., Guo H.S. (2016). Host-induced gene silencing of target gene in fungal cells confers effective resistance to cotton wilt disease pathogen *Verticillium dahliae*. Mol. Plant.

[18] Nury H., Dahout-Gonzalez C., Trezeguet V., Lauquin G., Brandolin G., Pebay-Peyroula E. (2005). Structural basis for lipid-mediated interactions between mitochondrial ADP/ATP carrier monomers. FEBS Lett..

[19] Klingenberg M. (1989). Molecular aspects of the adenine nucleotide carrier from mitochondria. Arch. Biochem. Biophys..

[20] Fiore C., Le S.A., Roux P., Schwimmer C., Dianoux A.N.F., Gjm L., Brandolin G., Pv V., Trezeguet V. (1998). The mitochondrial ADP/ATP carrier: Structural, physiological and pathological aspects. Biochimie.

[21] Pebay-Peyroula E., Dahout-Gonzalez C., Kahn R., Trezeguet V., Lauquin G.J., Brandolin G. (2003). Structure of mitochondrial ADP/ATP carrier in complex with carboxyatractyloside. Nature.

[22] Hatanaka T., Kihira Y., Shinohara Y., Majima E., Terada H. (2001). Characterization of loops of the yeast mitochondrial ADP/ATP carrier facing the cytosol by site-directed mutagenesis. Biochem. Biophys. Res. Commun..

[23] Ohkura K., Hori H., Shinohara Y. (2009). Role of C-terminal region of yeast ADP/ATP carrier 2 protein: Dynamics of flexible C-terminal arm. Anticancer Res..

[24] Nowara D., Gay A., Lacomme C., Shaw J., Ridout C., Douchkov D., Hensel G., Kumlehn J., Schweizer P. (2010). HIGS: Host-induced gene silencing in the obligate biotrophic fungal pathogen *Blumeria graminis*. Plant Cell.

[25] Pereira C., Chaves S., Alves S., Salin B., Camougrand N., Manon S., Sousa M.J., Corte-Real M. (2010). Mitochondrial degradation in acetic acid-induced yeast apoptosis: The role of Pep4 and the ADP/ATP carrier. Mol. Microbiol..

[26] Gigolashvili T., Geier M., Ashykhmina N., Frerigmann H., Wulfert S., Krueger S., Mugford S.G., Kopriva S., Haferkamp I., Flugge U.I. (2012). The *Arabidopsis* thylakoid ADP/ATP carrier TAAC has an additional role in supplying plastidic phosphoadenosine 5’-phosphosulfate to the cytosol. Plant Cell.

[27] Talbot D.A., Duchamp C., Rey B., Hanuise N., Rouanet J.L., Sibille B., Brand M.D. (2004). Uncoupling protein and ATP/ADP carrier increase mitochondrial proton conductance after cold adaptation of king penguins. J. Physiol..

[28] Gnipova A., Subrtova K., Panicucci B., Horvath A., Lukes J., Zikova A. (2015). The ADP/ATP carrier and its relationship to oxidative phosphorylation in ancestral protist *Trypanosoma brucei*. Eukaryot. Cell.

[29] Rehman L., Su X., Guo H., Qi X., Cheng H. (2016). Protoplast transformation as a potential platform for exploring gene function in *Verticillium dahliae*. BMC Biotechnol..

[30] Wang H.M., Lin Z.X., Zhang X.L., Chen W., Guo X.P., Nie Y.C., Li Y.H. (2008). Mapping and quantitative trait loci analysis of *Verticillium* wilt resistance genes in cotton. J. Integr. Plant Biol..

[31] Tian J., Zhang X., Liang B., Li S., Wu Z., Wang Q., Leng C., Dong J., Wang T. (2010). Expression of baculovirus anti-apoptotic genes *p35* and op-iap in cotton (*Gossypium hirsutum* L.) enhances tolerance to *Verticillium* wilt. PLoS ONE.

[32] Yang X., Ben S., Sun Y., Fan X., Tian C., Wang Y. (2013). Genome-wide identification, phylogeny and expression profile of vesicle fusion components in *Verticillium dahliae*. PLoS ONE.

[33] Bustin S.A., Benes V., Garson J.A., Hellemans J., Huggett J., Kubista M., Mueller R., Nolan T., Pfaffl M.W., Shipley G.L. (2009). The MIQE guidelines: Minimum information for publication of quantitative real-time PCR experiments. Clin. Biochem..

[34] Hoppenau C.E., Tran V.-T., Kusch H., Aßhauer K.P., Landesfeind M., Meinicke P., Popova B., Braus-Stromeyer S.A., Braus G.H. (2014). *Verticillium dahliae* VdTHI4, involved in thiazole biosynthesis, stress response and DNA repair functions, is required for vascular disease induction in tomato. Environ. Exp. Bot..

[35] Tzima A.K., Paplomatas E.J., Tsitsigiannis D.I., Kang S. (2012). The G protein beta subunit controls virulence and multiple growth- and development-related traits in *Verticillium dahliae*. Fungal Genet. Biol..

[36] Panwar V., McCallum B., Bakkeren G. (2013). Endogenous silencing of *Puccinia triticina* pathogenicity genes through in planta-expressed sequences leads to the suppression of rust diseases on wheat. Plant J..

[37] Ellendorff U., Fradin E.F., de Jonge R., Thomma B.P. (2009). RNA silencing is required for *Arabidopsis* defence against *Verticillium* wilt disease. J. Exp. Bot..

[38] Zhang Z., Song Y., Liu C.M., Thomma B.P. (2014). Mutational analysis of the Ve1 immune receptor that mediates *Verticillium* resistance in tomato. PLoS ONE.

[39] Mao Y., Cai W., Wang J., Hong G., Tao X., Wang L., Huang Y., Chen X. (2007). Silencing a cotton bollworm P450 monooxygenase gene by plant-mediated RNAi impairs larval tolerance of gossypol. Nat. Biotechnol..

[40] Obrepalska-Steplowska A., Wieczorek P., Budziszewska M., Jeszke A., Renaut J. (2013). How can plant virus satellite RNAs alter the effects of plant virus infection? A study of the changes in the *Nicotiana benthamiana* proteome after infection by peanut stunt virus in the presence or absence of its satellite RNA. Proteomics.

[41] Klionsky D.J., Nelson H., Nelson N., Yaver K. (1992). Mutations in the yeast vacuolar ATPase result in the mislocalization of vacuolar proteins. J. Exp. Biol..

[42] Clague M.J., Urbe S., Aniento F., Gruenberg J. (1994). Vacuolar ATPase activity is required for endosomal carrier vesicle formation. J. Biol. Chem..

[43] Klionsky D.J., Herman P.K., Emr S.D. (1990). The fungal vacuole: Composition, function, and biogenesis. Microbiol. Rev..

[44] Deckers-Hebestreit G., Schmid R., Kiltz H.H., Altendorf K. (1987). F0 portion of *Escherichia coli* ATP synthase: Orientation of subunit c in the membrane. Biochemistry.

[45] Van Walraven H.S., Scholts M.J., Lill H., Matthijs H.C., Dilley R.A., Kraayenhof R. (2002). Introduction of a carboxyl group in the loop of the F0 c-subunit affects the H^+^/ATP coupling ratio of the ATP synthase from *Synechocystis 6803*. J. Bioenerg. Biomembr..

[46] Salomon Y., Londos C., Rodbell M. (1974). A highly sensitive adenylate cyclase assay. Anal. Biochem..

[47] Klimpel A., Gronover C.S., Williamson B., Stewart J.A., Tudzynski B. (2002). The adenylate cyclase (BAC) in *Botrytis cinerea* is required for full pathogenicity. Mol. Plant Pathol..

[48] Piszkiewicz D., Tilley B.E., Rand-Meir T., Parsons S.M. (1979). Amino acid sequence of ATP phosphoribosyltransferase of *Salmonella typhimurium*. Proc. Natl. Acad. Sci. USA.

[49] Cho Y., Sharma V., Sacchettini J.C. (2003). Crystal structure of ATP phosphoribosyltransferase from *Mycobacterium tuberculosis*. J. Biol. Chem..

[50] Brenner M., Ames B.N., Vogel H.J. (1971). The histidine operon and its regulation. Metabolic Regulation.

[51] Goldberger R.F., Kovach J.S. (1972). Regulation of histidine biosynthesis in *Salmonella typhimurium*. Curr. Top. Cell. Regul..

[52] Voncken F., Gao F., Wadforth C., Harley M., Colasante C. (2013). The phosphoarginine energy-buffering system of *Trypanosoma brucei* involves multiple arginine kinase isoforms with different subcellular locations. PLoS ONE.

[53] Pereira C.A. (2014). Arginine kinase: A potential pharmacological target in trypanosomiasis. Infect. Disord. Drug Targets.

[54] Miranda M.R., Canepa G.E., Bouvier L.A., Pereira C.A. (2006). Trypanosoma cruzi: Oxidative stress induces arginine kinase expression. Exp. Parasitol..

[55] Liu T., Wang H., Kuang J., Sun C., Shi J., Duan X., Qu H., Jiang Y. (2015). Short-term anaerobic, pure oxygen and refrigerated storage conditions affect the energy status and selective gene expression in litchi fruit. LWT-Food Sci. Technol..

[56] Trezeguet V., Zeman I., David C., Lauquin G.J., Kolarov J. (1999). Expression of the ADP/ATP carrier encoding genes in aerobic yeasts; phenotype of an ADP/ATP carrier deletion mutant of *Schizosaccharomyces pombe*. Biochim. Biophys. Acta.

[57] Miura K., Inouye S., Sakai K., Takaoka H., Kishi F., Tabuchi M., Tanaka T., Matsumoto H., Shirai M., Nakazawa T., Nakazawa A. (2001). Cloning and characterization of adenylate kinase from *Chlamydia pneumoniae*. J. Biol. Chem..

[58] Claypool S.M., Oktay Y., Boontheung P., Loo J.A., Koehler C.M. (2008). Cardiolipin defines the interactome of the major ADP/ATP carrier protein of the mitochondrial inner membrane. J. Cell Biol..

[59] Hawke M.A., Lazarovits G. (1994). Production and manipulation of individual microsclerotia of *Verticillium dahliae* for use in studies of survival. Phytopathology.

[60] Isaac I. (1946). Verticillium wilt of sainfoin. Ann. Appl. Biol..

[61] Debode J., De Maeyer K., Perneel M., Pannecoucque J., De Backer G., Hofte M. (2007). Biosurfactants are involved in the biological control of *Verticillium microsclerotia* by *Pseudomonas* spp.. J. Appl. Microbiol..

[62] Zhang Y.L., Li Z.F., Feng Z.L., Feng H.J., Zhao L.H., Shi Y.Q., Hu X.P., Zhu H.Q. (2015). Isolation and functional analysis of the pathogenicity-related gene *VdPR3* from *Verticillium dahliae* on cotton. Curr. Genet..

[63] Santhanam P., Boshoven J.C., Salas O., Bowler K., Islam T., Keykha Saber M., van den Berg G.C., Bar-Peled M., Thomma B.P. (2016). Rhamnose synthase activity is required for pathogenicity of the vascular wilt fungus *Verticillium dahliae*. Mol. Plant Pathol..

[64] Novina C.D., Sharp P.A. (2004). The RNAi revolution. Nature.

[65] Kalantidis K., Psaradakis S., Tabler M., Tsagris M. (2002). The occurrence of CMV-specific short RNAS in transgenic tobacco expressing virus-derived double-stranded RNA is indicative of resistance to the virus. MPMI.

[66] Huang G., Allen R., Davis E.L., Baum T.J., Hussey R.S. (2006). Engineering broad root-knot resistance in transgenic plants by RNAi silencing of a conserved and essential root-knot nematode parasitism gene. Proc. Natl. Acad. Sci. USA.

[67] Schwind N., Zwiebel M., Itaya A., Ding B., Wang M.B., Krczal G., Wassenegger M. (2009). RNAi-mediated resistance to Potato spindle tuber viroid in transgenic tomato expressing a viroid hairpin RNA construct. Mol. Plant Pathol..

