# Supplementary material for: AAC as a Potential Target Gene to Control Verticillium dahliae
Source: Genes (Basel). 2017 Jan 10;8(1):25. doi: 10.3390/genes8010025 (PMC5295020; doi:10.3390/genes8010025)
Supplement: Supplementary file 1 [file genes-08-00025-s001.docx]

Supplementary Materials: *AAC* as a Potential Target Gene to Control *Verticillium dahliae*

Xiaofeng Su, Latifur Rehman, Huiming Guo, Xiaokang Li, Rui Zhang and Hongmei Cheng


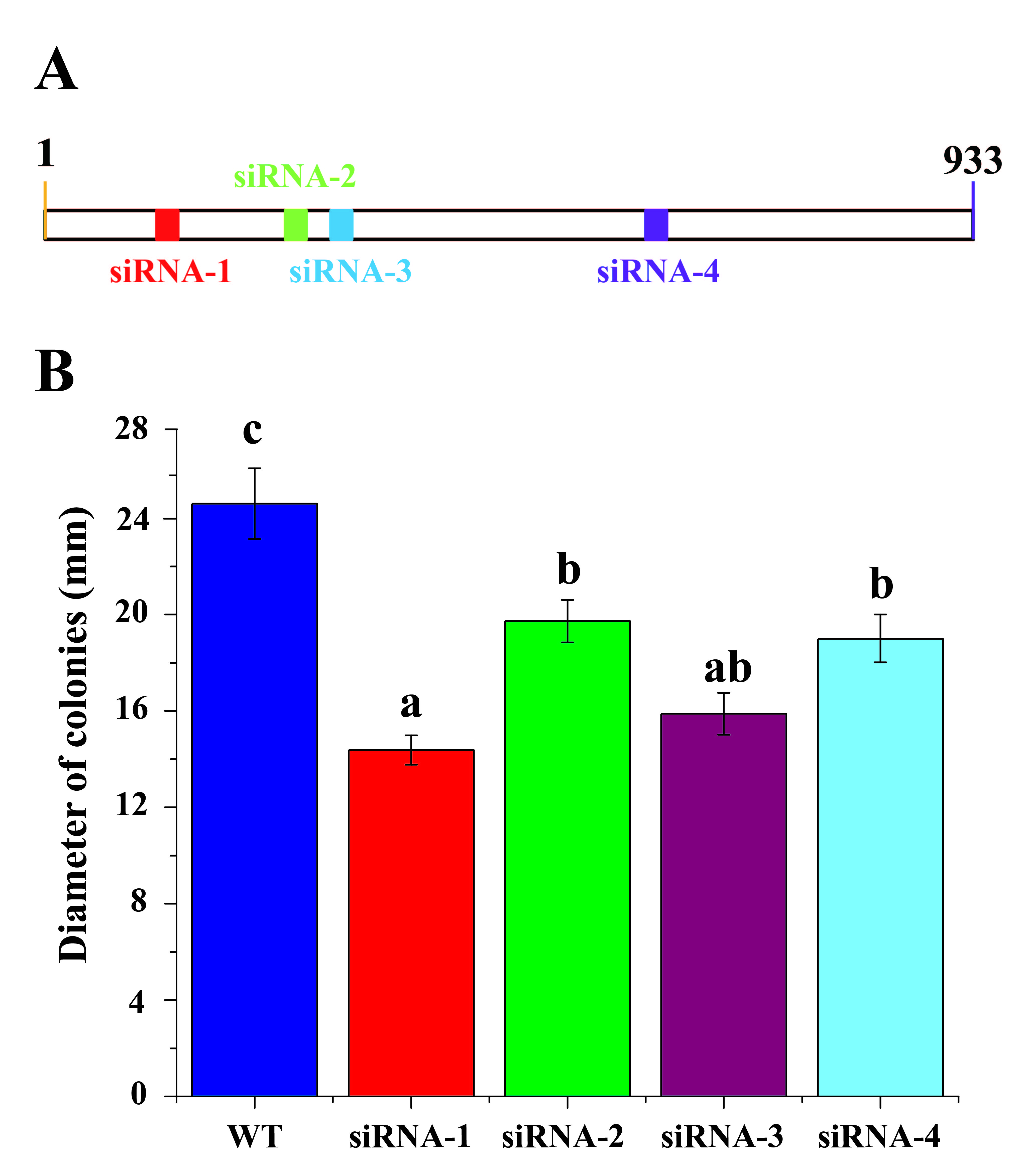


**Figure S1.** Position of siRNAs designed from different regions of the *VdAAC* gene of *V. dahliae* and colony diameter in different RNAi-treated groups. (**A**) Position of siRNAs along the VdAAC gene. siRNAs were designed and synthesized by Oligobio, Beijing, China; (**B**) Colony diameters of control and siRNA groups observed 2 weeks after transformation on PDA agar plates. The bars with different letters are significantly different (*p <* 0.05), based on the Duncan’s multiple range test.


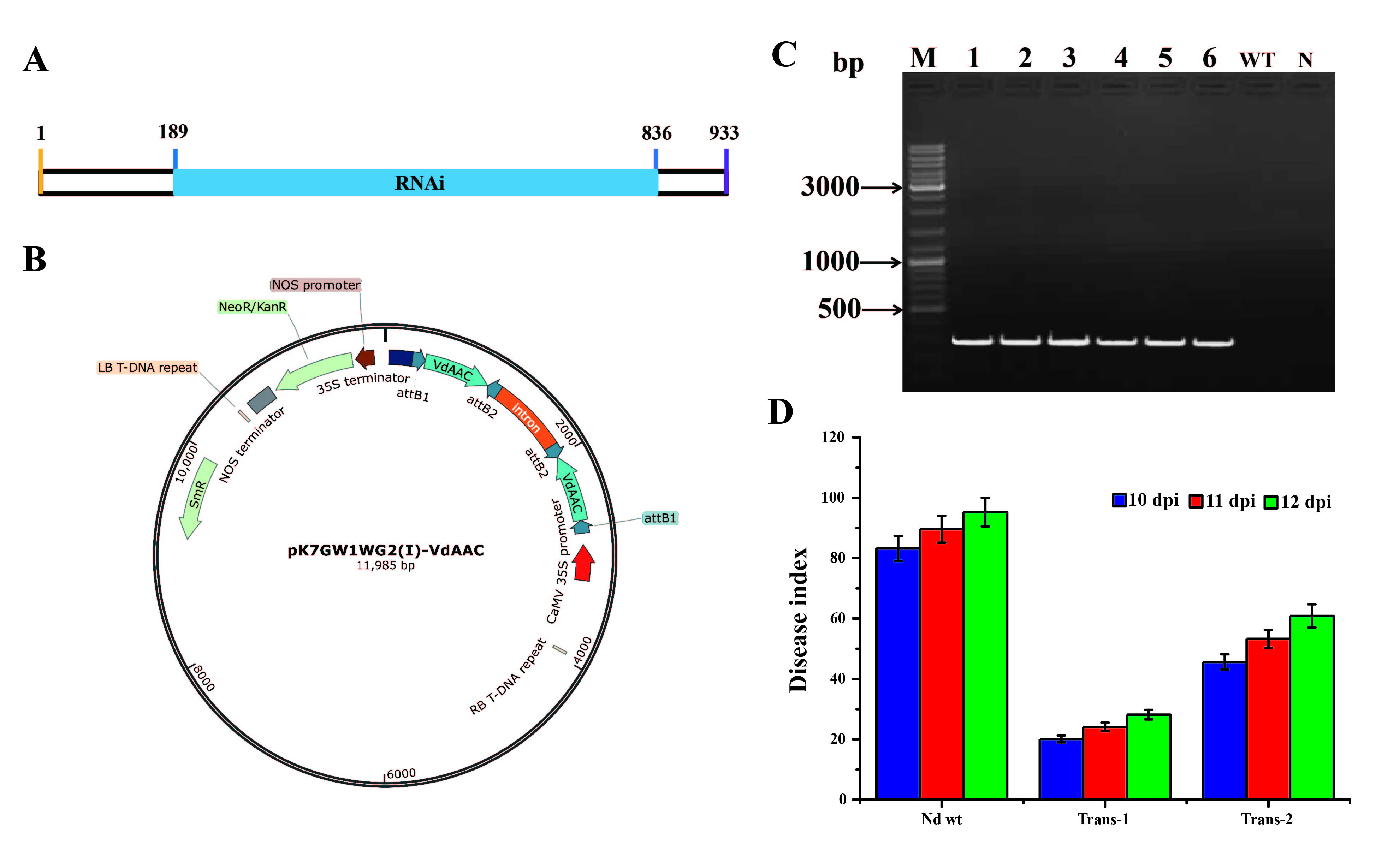


**Figure S2.** Evaluation of resistance for wild-type (Nb wt) and transgenic *N. benthamiana* against *V. dahliae*. (**A**) Region (189–836 bp) of *VdAAC* gene was amplified and cloned into pK7GW1WG2(I) by LR recombination reaction. Numbers indicate nucleotide positions; (**B**) Schematic representation of the pK7GWIWG2(I)-VdAAC construction containing the sense and antisense partial ORF of VdAAC; (**C**) Confirmation of transgenic plants transformed with pK7GWIWG2(I)-VdAAC by PCR. The Nb wt seedling served as the negative control; (**D**) Disease index for seedlings from 10 to 12 days post inoculation with *V. dahliae*.


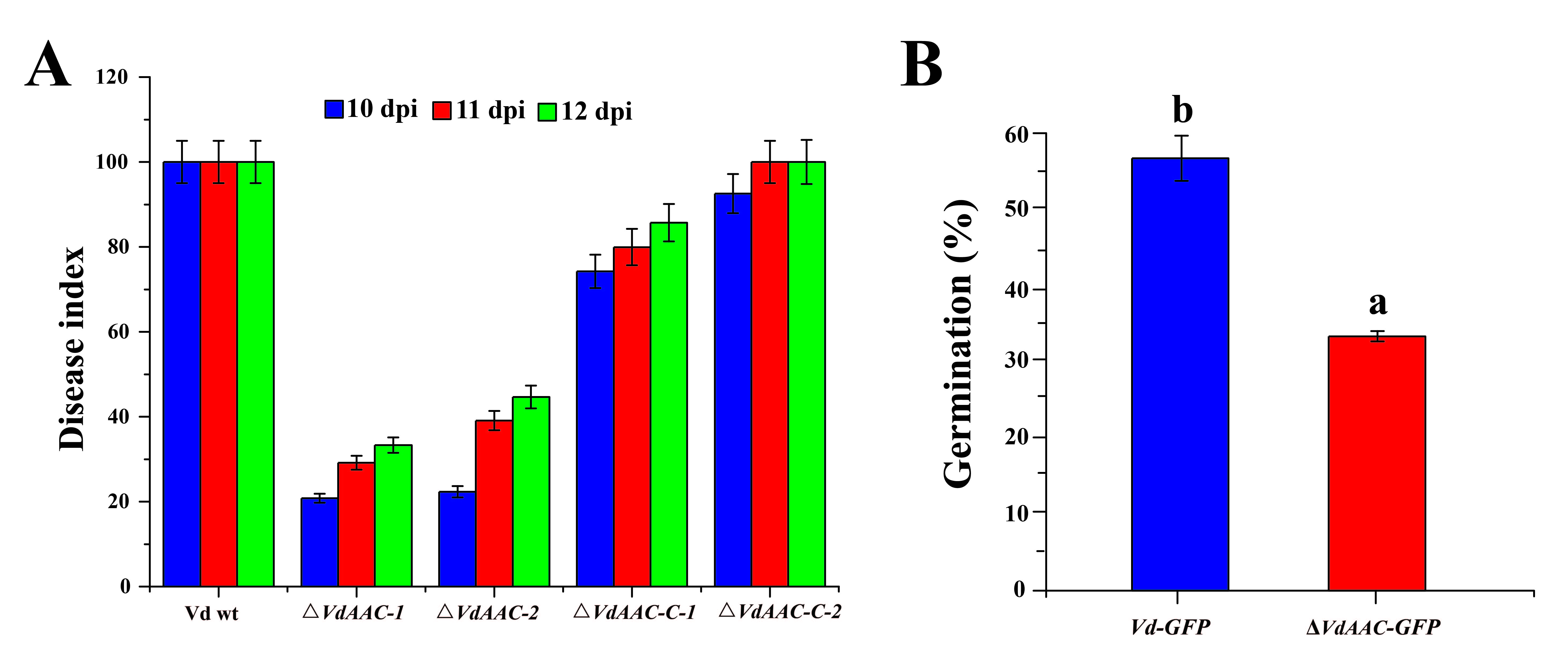


**Figure S3.** Virulence and germination analysis of mutants and wild-type *V. dahliae* (Vd wt). (**A**) Disease index for *N. benthamiana* seedlings at 10 to 12 dpi with ΔVdAAC, ΔVdAAC-C and Vd wt; (**B**) Percentage germination of conidia produced by Vd-GFP or ΔVdAAC-GFP after 48 h on PDA. Duncan’s multiple range test was applied to determine significant differences among the treatment groups (*p <* 0.05) indicated by different letters (a, b).
